# DTUMOS, digital twin for large-scale urban mobility operating system

**DOI:** 10.1038/s41598-023-32326-9

**Published:** 2023-03-29

**Authors:** Hyeokju Yeon, Taebum Eom, Kitae Jang, Jiho Yeo

**Affiliations:** 1 Department of Big Data Application, Hannam University, Daejeon, 34051 Korea; 2 KAIST, The Cho Chun Shik Graduate School of Mobility, Daejeon, 34051 Korea

**Keywords:** Civil engineering, Computational science, Computer science

## Abstract

The advancement of digital twin technology has significantly impacted the utilization of virtual cities in the realm of smart cities and mobility. Digital twins provide a platform for the development and testing of various mobility systems, algorithms, and policies. In this research, we introduce DTUMOS, a digital twin framework for urban mobility operating systems. DTUMOS is a versatile, open-source framework that can be flexibly and adaptably integrated into various urban mobility systems. Its novel architecture, combining an AI-based estimated time of arrival model and vehicle routing algorithm, allows DTUMOS to achieve high-speed performance while maintaining accuracy in the implementation of large-scale mobility systems. DTUMOS exhibits distinct advantages in terms of scalability, simulation speed, and visualization compared to current state-of-the-art mobility digital twins and simulations. The performance and scalability of DTUMOS are validated through the use of real data in large metropolitan cities including Seoul, New York City, and Chicago. DTUMOS’ lightweight and open-source environment present opportunities for the development of various simulation-based algorithms and the quantitative evaluation of policies for future mobility systems.

Digital twin technology, which involves the creation of virtual representations of physical entities in cyberspace, has brought about innovative changes in various industries. Digital twins are being utilized for a range of purposes including smart manufacturing^1–7^, product quality and anomaly detection^8–10^, equipment behavior design^11,12^, and system management and control^13–15^. The rapid development of digital twin technology, which initially emerged in the manufacturing sector, has transformed and expanded the ways in which virtual reality is used in the fields of smart cities and transportation. While traffic simulations such as VISSIM^16^, AIMSUN^17^, SUMO^18^, and MATSim^19^ predated the concept of digital twins, the convergence of advanced digital twin technology with traffic simulation has demonstrated the potential for various innovative approaches.

The new digital twin technology has expanded beyond traffic congestion and flow analysis and signal optimization^20^ to (1) the training algorithms of autonomous vehicles^21–24^, (2) planning and managing transportation infrastructures^15,25^, and (3) the operation of transport systems and mobility services. Especially the development of autonomous vehicles, mobile devices, the internet of things, and citizens’ needs for new transportation systems accelerate the emergence of new and inventive mobility systems. In addition to traditional taxi and public transportation systems (e.g., subways and buses), various mobility-on-demand services such as Uber, Grab, and Lift, demand responsive transit, food delivery, and urban air mobility^26^ have emerged over the past decade. A digital twin can directly contribute greatly to the development and settlement of these new mobility systems. It can be used to develop and modify various algorithms necessary for the operation of mobility services. Innumerable bold and uncertain attempts that cannot be carried out in the real world can be made freely in the digital twin. Services that have not yet been commercialized, such as urban air mobility, can also be tested before being implemented and operated.

There are a few studies that mainly focus on the digital twin for mobility systems. Some studies directly implemented digital twins^27–30^, and other studies applied and modified existing traffic simulations^31,32^. However, most of the research focused on implementing mobility services of a prototype with a small number of vehicles, and the case study was also limited to a mobility system operated in small and local areas. To the authors’ knowledge, Amodeus^31^ is the most advanced digital twin for the mobility system. Compared with other existing studies, Amodeus has large scalability and is an open-source software based on MATSim. Various scenarios have been established for the operation of mobility systems in plenty of cities (Berlin, Santiago, Tel Aviv, New york city, Chicago)^27,33,34^.

Although continuous attempts and research have led to significant advancements in the capabilities of digital twins for urban mobility systems, there are still challenges that need to be addressed. First, the mobility digital twin must have greater scalability. Since various mobility services are provided in metropolitan areas beyond a single city, the mobility digital twin should be implemented in a wider spatial and temporal range. Second, simulation speed and visualization performance should be improved. Even when the scale is expanded to the metropolitan level, and tens of thousands of vehicles are being operated, it is essential to provide smooth visualization of vehicle movements and passenger behaviors. Lastly, the digital twin for mobility systems should be able to interact with various machine and deep learning algorithms to enhance its own performance.

To address these challenges, we propose DTUMOS, a Digital Twin for Urban Mobility Operating System, which implements a wide range of mobility systems in large-scale metropolitan cities. The research contributions and key strengths of DTUMOS in comparison to existing studies are as follows: (1) Scalability is significantly expanded and verified through the implementation of a large-scale mobility system in the metropolitan areas of Seoul, Chicago, and New York, where more than 20,000 vehicles are operated; (2) The proposed digital twin framework is enhanced by using learning-based models and optimization techniques, and we found that incorporating A.I. with the digital twin can greatly increase its speed and accuracy (i.e., its similarity with reality); (3) Through the direct implementation of various mobility services, multiple case studies were conducted to diagnose the problems of current mobility services, suggest alternatives, and evaluate the quantitative effectiveness of these alternatives.

## Methods

### Framework of digital twin for urban mobility operating system

It is crucial to clearly define the functionalities of the proposed mobility digital twin. DTUMOS serves as a test bed and playground for testing various mobility systems, rather than monitoring existing systems in real time. In DTUMOS, users should be able to flexibly modify the type of mobility system, dataset, and operation algorithms and strategies. Furthermore, the performance of the mobility system should be quantitatively assessed.

The essential aspect of mobility digital twins is to maintain a lightweight model while minimizing the discrepancy between the digital twin and reality. The core requirements of digital twins vary depending on their intended purpose. For instance, in digital twins used for training algorithms in autonomous vehicles, rendering quality is a critical factor because the larger the difference between reality and the rendering quality in the digital twin, the more significantly the performance of the self-driving car in the real world is impacted. Conversely, in digital twins for large-scale mobility systems, a significant number of vehicles and passengers should be represented, and their behaviors must be simulated in a manner that closely mirrors the real world.

**Figure 1:**
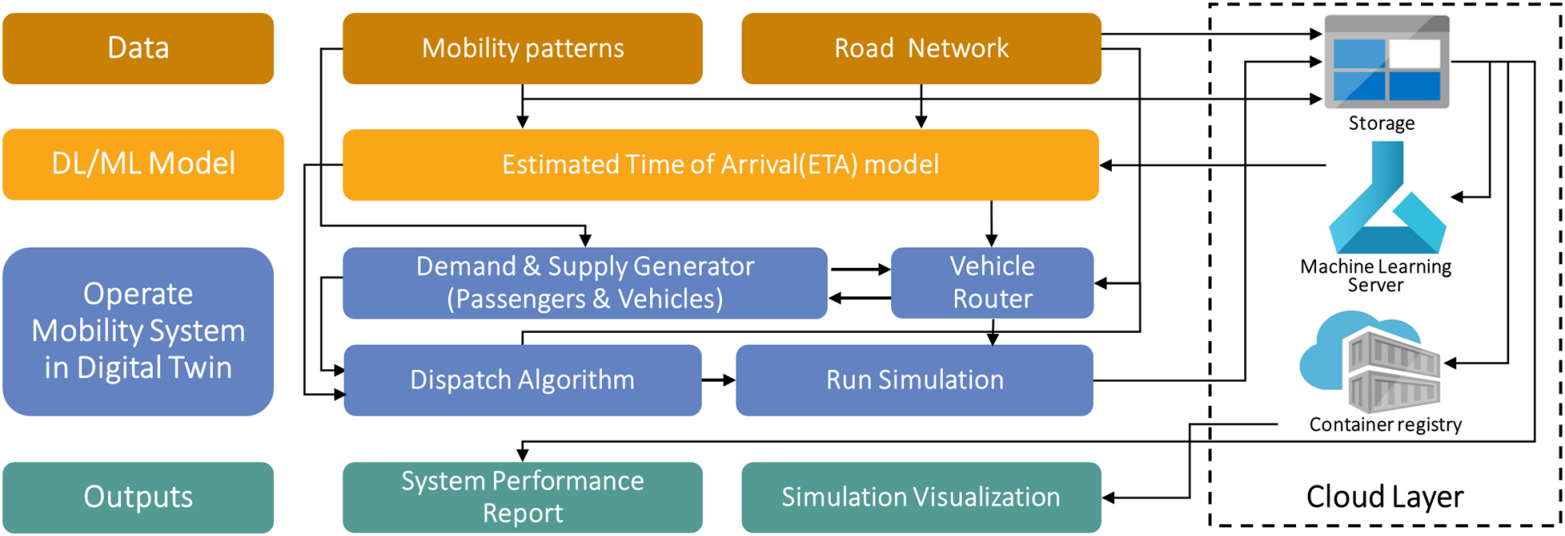
Illustration of the framework of DTUMOS.

In this manner, Fig. 1 illustrates the framework of the proposed DTUMOS. The framework consists of four parts: (1) Data; (2) A.I. and Machine learning models; (3) Operate mobility system; (4) Outputs. First, real-world data is the most fundamental element for the accurate representation of the physical world within mobility digital twins. This data encompasses usage patterns of passengers and goods, including information such as boarding and disembarkment locations and times. Historical mobility data, such as taxi trip records, smart card data, mobile phone cellular signaling data, and delivery and logistics data, can be utilized as input data. Additionally, the road network is utilized for routing vehicles and predicting travel times. In the study, we utilized OpenStreetMap^35^, a widely available open-source tool that can be applied to various cities worldwide, to serve this purpose.

The second component of the proposed framework includes the use of deep learning or machine learning models to further enhance the precision of the digital twin. The accurate calculation of travel time between origins and destinations is crucial for achieving a high degree of similarity between the digital twin and reality. The temporal and spatial characteristics of travel, such as traffic congestion, peak hours, and day-of-week and time-of-day information, must be considered in order to achieve this goal. To address this issue, we propose a novel framework that corrects the locations and travel times of vehicles. Utilizing deep learning techniques, an Estimated Time of Arrival (ETA) model was trained and applied to improve the accuracy and simulation speed of the DTUMOS. Additionally, while not included in the study, a travel demand prediction model or a mode choice model to assist users in selecting transportation options can also be included in this component.

The third part is the process of operating a mobility system in the digital twin. Based on historical trip records, supply and demand can be generated exactly as in reality, and user customization (e.g., spatial and temporal randomness, increase/decrease in demand and supply) can also be applied. After determining the distributions of passengers and vehicles, a dispatch algorithm that matches passengers and vehicles is required, and the simulation is running as dispatch, and vehicle routing are operated sequentially. The Open Street Route Machine (OSRM) is utilized for routing vehicles, which is open source and fast.

Finally, the DTUMOS generates simulation visualizations and performance reports as its final outputs. The performance report serves as a quantitative evaluation of the mobility service after the virtual simulation of the mobility service is completed in DTUMOS. The system performance report includes sections on level of service (LOS), vehicle operation information, and spatial analysis. The LOS section provides information on passenger waiting times and the number of failed requests. The criteria for failed requests should be established in advance and in this study, it was assumed that a dispatch failure occurs when a vehicle is not assigned or a passenger has to wait for more than 30 min. The vehicle operation information section displays the number of empty and in-service vehicles as well as the number of passengers waiting for dispatch by time, providing an intuitive understanding of whether the number of operating vehicles is sufficient or not. Lastly, in the spatial distribution section, the pick-up and drop-off locations of passengers and the locations where the failure occurred are visualized by time and region.

The cloud layer is not essential but useful in the framework. In the study, DTUMOS is implemented by utilizing cloud computing, Microsoft Azure. All input data and simulation results are stored in Azure storage, and the ETA model is trained on Azure Machine Learning Server. Finally, the simulation result is built as a docker image, and a large-scale mobility system can be visualized smoothly and seamlessly through continuous communication with a cloud layer.

### Estimated time of arrival (ETA) model

The input features for training the ETA model in Eq. (1) consists of the latitude and longitude coordinates of origins and destinations, the linear distance between origins and destinations, departure time, the day of the week, public holidays, and the administrative district of origins and destinations. To train the ETA model, 63,14373 samples of historical taxi pick-up and drop-off data for six months from January to June 2018 in the Seoul Metropolitan Area were used. 60% of the data was set as a train, 20% as a validation, and 20% as a test.

We conducted cross-validation on various machine learning and deep learning models to predict ETA. In this study, several gradient Boosting trees (e.g., XGBoost, CatBoost, LightGBM) and deep learning models, TabNet, which is widely used for tabular data, were adopted as baselines, and CatBoost was finally selected as the best model as seen in Table 1. Two evaluation metrics, the Mean Absolute Percentage Error (MAPE) and the Root Mean Squared Error (RMSE), were used to evaluate the performance of the ETA models. Since small-value observations make the MAPE value too large, so we filtered out samples less than ten from the MAPE calculation according to previous studies^36^. We utilized a grid-search and cross-validation methods that compare the performance of the model with all combinations of hyper-parameters^37^. The optimal hyper-parameters are a learning rate of 0.3, depth of the tree of 10, number of iterations of 1000, and coefficient at the L2 regularization term of 0.2.

**Table 1:** Computation time (min) by changing the number of vehicles and requests per day.

Performance	Linear model	XGBoost	TabNet	LightGBM	CatBoost
RMSE	120.05	6.82	4.42	4.32	4.16
MAPE	373.03	30.83	30.48	29.18	27.93

DTUMOS enables the implementation of extremely large-scale mobility systems at the metropolitan level.

### Dispatch algorithm

In all mobility services implemented as case studies in this study, passengers and vehicles were matched one-to-one. Then, the dispatch algorithm is the assignment problem where a group of call requests is assigned to a set of vehicles. This problem can be solved by using a combinatorial optimization technique. The call requests are matched every minute with the vehicles, and the objective function is designed so that the sum of the waiting times for all passengers is minimized.

Consider the problem of assigning *m* call requests to *n* vehicles (one vehicle to one request). Let $C_{ij}$ be the cost of assigning *i*th request to the *j*th vehicle and $x_{ij}$ represents the assignment of *i*th request to the *j*th vehicle. Then, $x_{ij}$ is formulated as follows:

1 $$\begin{aligned} x_{ij} = \left\{ \begin{matrix} 1, &{} \text {if} \ i{th} \ \text {request} \ \text {is} \ \text {assigned} \ \text {to} \ j{th} \ \text {vehicle} \\ 0, &{} \text {if} \ i{th} \ \text {request} \ \text {is} \ \text {not} \ \text {assigned} \ \text {to} \ j{th} \ \text {vehicle} \\ \end{matrix}\right. \end{aligned}$$

The assignment model can be written as follows:

2 $$\begin{aligned} Minimize \ Z = \sum _{i=1}^{m}\sum _{j=1}^{n}C_{ij}x_{ij} \end{aligned}$$

Subject to the constraints,

3 $$\begin{aligned} \sum _{i=1}^{m}x_{ij} = 1, \ i=1,2,...,n \end{aligned}$$

and

4 $$\begin{aligned} \sum _{j=1}^{n}x_{ij} = 1, \ j=1,2,...,m \end{aligned}$$

Google OR-tools^38^ was used as a solver, which is open-source and is easily applicable to DTUMOS.

On the other hand, in the case of the Seoul Metropolitan Area as shown in Table 3, due to a large number of vehicles and passengers (exceeding 10,000 and 50,000 respectively), finding the global optimal solution becomes computationally expensive. To address this scalability issue, a greedy algorithm was implemented. The vehicles were dispatched to the nearest passenger based on the First-In-First-Out (FIFO) method. It should be noted that optimizing the dispatch algorithm and ETA model was not the primary focus of this study. The aim of this study is to present a framework for creating a more accurate digital twin by integrating ETA models and mobility simulations.

## Results

### Implementation of DTUMOS

The detailed process of the simulation operation is further explained in the pseudo-code below in addition to the illustrated framework in Fig. 1. As shown in Algorithm 1, the input variables include the “vehicle_operation”, containing information on the status and location of vehicles, and the “passenger”, containing information on the call request time and location of passengers. Upon inputting these corresponding variables, a loop statement is executed based on time. In the loop statement, the dispatch algorithm is utilized to match vehicles and passengers on a minute-by-minute basis. The algorithm then calculates the optimal route and estimated travel time for the matched vehicle to reach the passenger. Vehicle router and ETA models are utilized in this step. Finally, the status and location of both the vehicle and passenger are continuously updated during the loop, and the final output of the simulation is saved as an output file.

This framework is noteworthy in that it can respond to changes in various mobility services and algorithms in a flexible manner. When the city operating the mobility service or the type of objects utilizing the service (e.g., passengers, goods, foods, etc.) are changed, it can be implemented simply by modifying the input data. Furthermore, individual modules and functions can be modified if the service type or operating algorithm changes. For instance, to change the dispatch algorithm that matches users and vehicles, the *dispatch_vehicles* function in 2–c can be altered. To determine the optimal vehicles’ routes and travel time by considering updated traffic information, even if it requires a trade-off with speed and incurs additional expenses, the modules in 2–d and 2–g in Algorithm 1 can be altered to Google API or other paid vehicle routing APIs. As will be described in more detail in a later section, in this study, various mobility services were implemented in various cities using this flexible and concise structure.
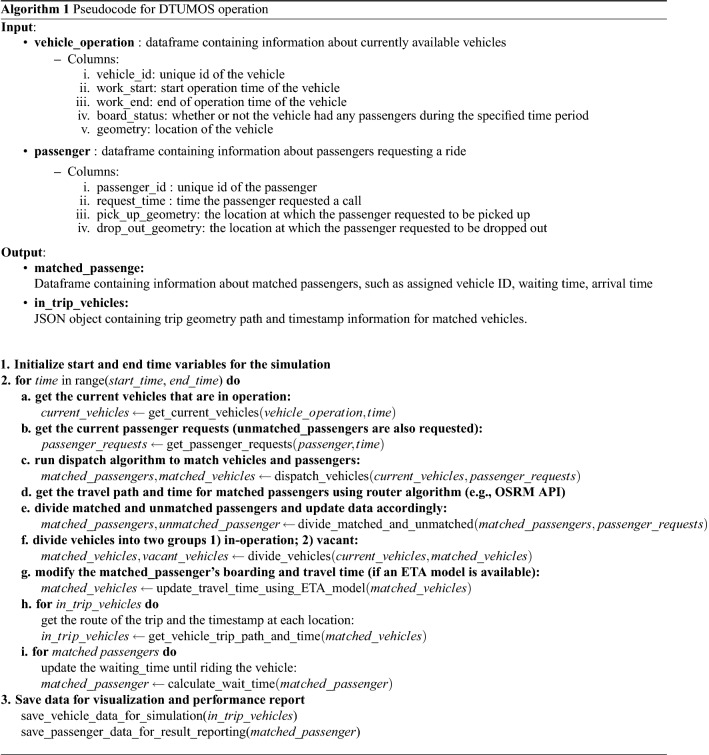


**Figure 2:**
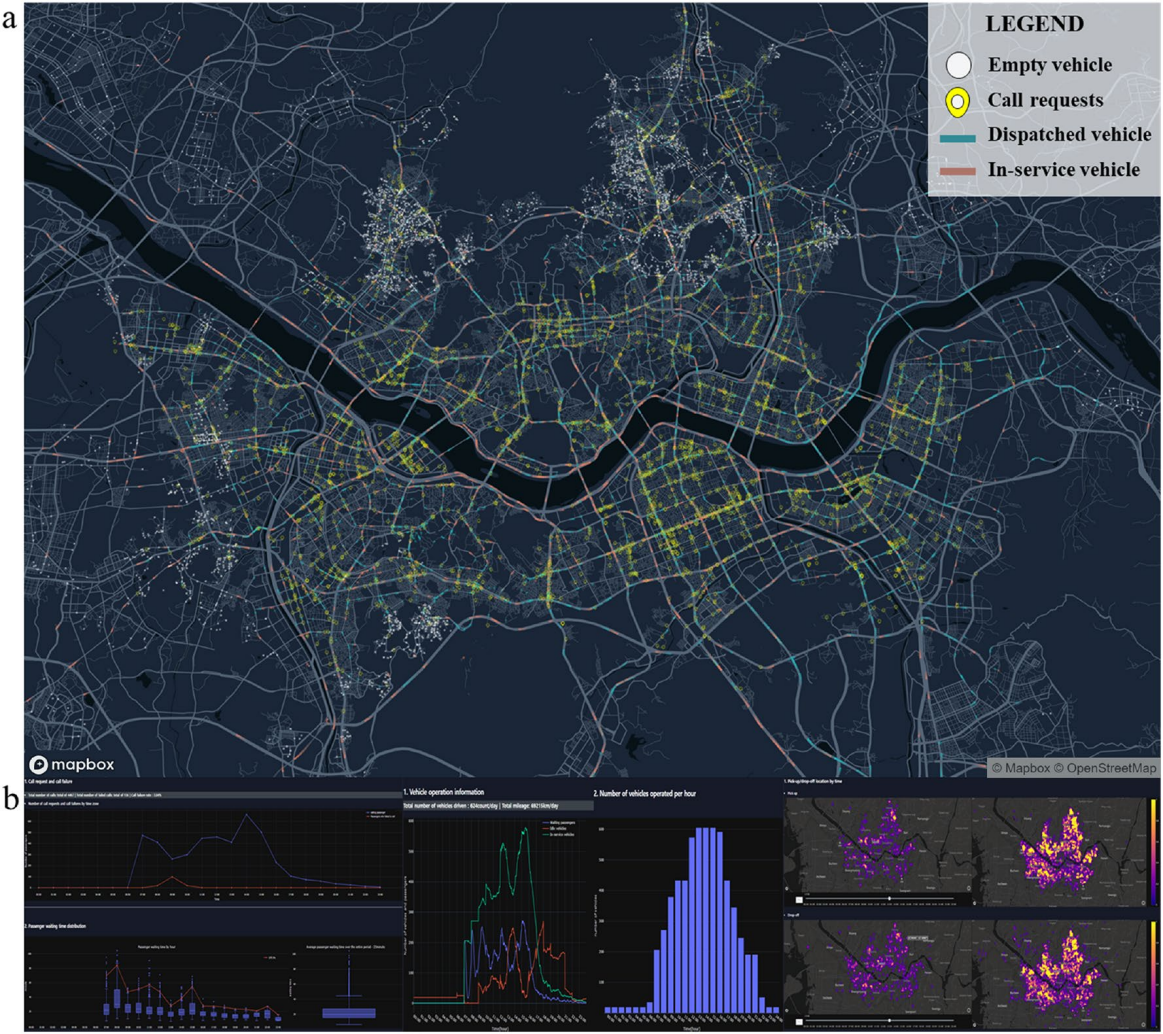
Final implementation of DTUMOS in Seoul Metropolitan Area. (**a**) a simulation visualization using deck.gl framework (**b**) a system performance report containing level of service (LOS) of the system, vehicle operation information, and spatial analysis. See a simulation visualization and a system performance report in a web browser. The map is created using Mapbox GL JS v.2.12.0 (https://www.mapbox.com/).

An example of the DTUMOS implementation for a taxi service in the Seoul Metropolitan Area is presented in Fig. 2. The final results consist of two components: a simulation visualization (Fig. 2a) and a system performance report (Fig. 2b). The visualization serves as a crucial indicator for evaluating the similarity between the digital twin and reality. By displaying the system’s operation in time and space, potential issues can be identified and improvements to the mobility system can be proposed. In Fig. 2a, the locations of empty vehicles and passengers are represented by white and yellow points, respectively. The movement of vehicles can be better observed as the vehicles in operation are visualized as paths rather than points. The vehicles assigned to dispatched passengers are represented as blue paths, while the vehicles carrying passengers are represented as orange paths. We utilized deck.gl, an open-source visualization framework specialized for data-heavy visual analysis^39^. deck.gl enables users to easily create complex visualizations by providing a variety of independent layers. In particular, the TripLayer feature renders animated paths that are suitable for representing the movement of a large number of vehicles smoothly. For further and detailed information, see a simulation visualization and a system performance report that are the outputs of implementing mobility systems in Seoul Metropolitan Area.

### Learning-based algorithm for improving the accuracy and reliability of DTUMOS

Calculating the precise estimated time of arrival (ETA) of vehicles is a key element to realistically simulating vehicles’ movements and efficiently constructing the dispatch algorithm that assigns vehicles to passengers. It is, therefore, one of the most significant factors in determining the accuracy and reliability of the mobility digital twin. In DTUMOS, a novel method for modeling vehicles’ movements with high accuracy and fast calculation speed was presented by training the ETA model directly and calibrating the vehicle router through the model. Consider that ${\textbf {p}}$ is the location vector of the vehicle and ${\textbf {t}}$ is the time vector, which represents the positions and time information of a single vehicle. Then ${\textbf {p}}$ and ${\textbf {t}}$ is represented as:

5 $$\begin{aligned} {\textbf {p}} = \begin{Bmatrix} {p_1, p_2, p_3,\ldots , p_{n-1}, p_n} \end{Bmatrix} \quad and \quad {\textbf {t}} = \begin{Bmatrix} {t_1, t_2, t_3,\ldots , t_{n-1}, t_n} \end{Bmatrix} \end{aligned}$$

,where *n* is the number of points the vehicle router makes when moving a vehicle from $p_1$ to $p_n$. Finally, the ETA model, *F*, predicts the travel time, ${\hat{y}}$, from origin to destination of the vehicle as:

6 $$\begin{aligned} {\hat{y}} = F(p_1,p_n,{\textbf {x}}) \end{aligned}$$

**x** is other input features of the ETA model, which are described in detail in the method section. Then, the time vector, ${\textbf {t}}$, is calibrated and updated to $t^{\prime }$ as follows:

7 $$\begin{aligned} t_{i}^{\prime } - t_{j}^{\prime } =(t_{i} - t_{j}) \cdot \frac{{\hat{y}}}{t_n-t_1},\ \forall i,j \end{aligned}$$

Calibrating travel time in this manner offers a faster and more accurate solution compared to correcting travel time through Direction API, assuming the ETA model is well-trained. Table 2 compares the performance and speed of the Direction API, Naver, Tmap, and the learning-based ETA model with Naive OSRM. Naver and Tmap are companies that possess their own maps and navigation and provide Direction APIs. The evaluation was conducted by sampling 20% of the taxi pick-up and drop-off data in the Seoul Metropolitan Area in 2018. Google’s Direction API was not considered due to its unavailability in South Korea. To assess accuracy, Root Mean Squared Error (RMSE) and Mean Absolute Percentage Error (MAPE) were used as evaluation metrics, and the results indicate that Tmap API performs best in terms of accuracy. However, the difference in accuracy between the model utilizing Direction APIs and the learning-based ETA is minimal in comparison to the significant difference in computation speed. Based on a single sample, the learning-based ETA model computes more than 100 times faster than Direction APIs, and the computation time increases noticeably as ETA for a larger number of samples is calculated.

**Table 2:** Performance comparison of Estimated Time of Arrival (ETA): The proposed learning-based ETA model is incredibly fast compared to others while maintaining similar accuracy.

ETA algorithm	Accuracy			Computation time ($\mu$s)		
	RMSE (min)	MAPE (%)		1 sample	10 samples	100 samples
Naive OSRM	13.39	57.10		4.45	35.72	166.69
Naver API	7.10	27.97		122.81	698.72	7398.60
Tmap API	7.27	33.28		955.17	1269.59	13750.60
Learning-based ETA	4.94	21.35		0.98	1.08	1.73

We can improve the efficiency of the dispatch algorithm by utilizing the learning-based ETA model as well. The dispatch algorithm, as explained in the method section, involves calculating the cost matrix, $C_{ij}$, between all vehicles and call requests, and identifying the optimal passenger-vehicle pairs that minimize the total cost. If the cost is defined as the travel time between the vehicle and a call request, the ETA algorithm can be employed to calculate $C_{ij}$. As a result, an extremely large-scale mobility system can be implemented with efficient computational performance, which will be elucidated in a subsequent section.

### Scalability and extensibility of DTUMOS

In this section, the experimental validation of scalability and extensibility is carried out. It is imperative to highlight the significance of scalability and extensibility in the context of a mobility digital twin and to enumerate the various aspects that must be taken into consideration to validate the high scalability and extensibility of the DTUMOS. The experiments were conducted in two parts. Firstly, the performance of DTUMOS was compared with the previous state-of-the-art mobility simulation, AmoDeus^31^. Secondly, the extensibility of DTUMOS was demonstrated through the implementation of various types of mobility services in different metropolitan cities.

DTUMOS features a lightweight framework that is crucial for high scalability. Specifically, the calculation of vehicle routes and the cost matrix (e.g., travel time) between passengers and vehicles requires a significant amount of computational resources. As previously noted, DTUMOS significantly improves the efficiency of vehicle routing and dispatch through the integration of an open street map-based router algorithm with a learning-based ETA model. To verify this, we compared the implementation time for a large-scale mobility system in DTUMOS to that of Amodeus by increasing the number of vehicles operating in the Seoul Metropolitan Area from 10,000 to 20,000, and the number of requests per day from 50,000 to 400,000. The computation time was calculated through a series of simulation runs on a computer with 128 GB of RAM and a 3.4 GHz processor.

As presented in Table 3, DTUMOS enables the implementation of large-scale mobility systems at the metropolitan level, outperforming the state-of-the-art model, Amodeus, by more than twice the speed. The performance difference becomes more pronounced as the scale of the mobility system increases. Furthermore, the mobility system with more than 20,000 vehicles and more than 100,000 passengers could only be implemented using DTUMOS, as the state-of-the-art model was unable to handle such a large scale. To the best of the authors’ knowledge, this is the first attempt by DTUMOS to implement such a large-scale mobility system.

**Table 3:** Computation time (min) by changing the number of vehicles and requests per day.

Simulation size	Computation time (min)	
	AMODeus^31^	DTUMOS
10,000 vehicles and 50,000 requests per day	126.05	59.17
20,000 vehicles and 50,000 requests per day	379.03	58.33
20,000 vehicles and 100,000 requests per day	Not applicable	106.15
20,000 vehicles and 200,000 requests per day	Not applicable	157.36
20,000 vehicles and 400,000 requests per day	Not applicable	173.11

DTUMOS enables the implementation of extremely large-scale mobility systems at the metropolitan level.

**Figure 3:**
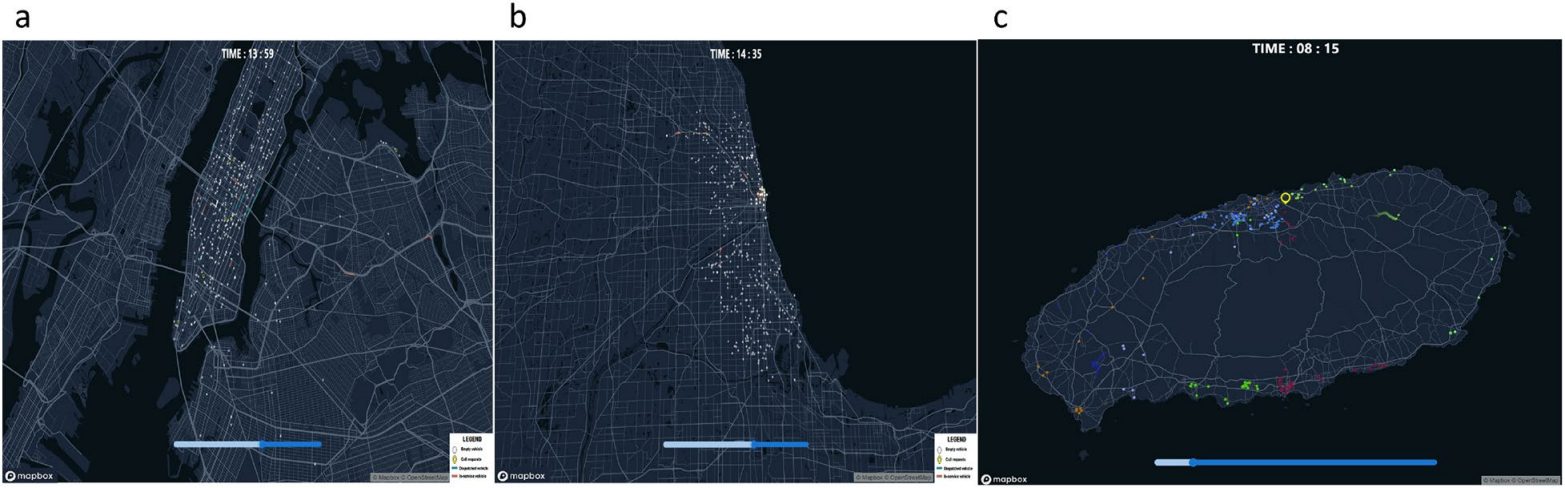
High extensibility of DTUMOS: (**a**) Taxi service in New York City; (**b**) Taxi service in Chicago; (**c**) Logistics Delivery Service in Jeju island in South Korea. The map is created using Mapbox GL JS v.2.12.0 (https://www.mapbox.com/).

Since all modules in DTUMOS are developed based on open-source software and each module is concisely classified according to its function presented in Algorithm 1, DTUMOS can be easily applied to various cities and diverse mobility services around the world. In order to show the incredible extensibility of DTUMOS, various examples are provided in various types of mobility services in different metropolitan cities as presented in Table 4. Large metropolitan cities, such as Seoul, New York, and Chicago, have implemented large-scale mobility services, encompassing a range of services such as depot-based delivery and many-to-many pickup and delivery, in addition to traditional taxi services. See Fig. 3 for examples of the implementation. Furthermore, as the visualization framework using deck.gl provides powerful 3D visualization, urban air mobility services (UAM), and underground mobility services such as hyperloop can also be implemented without difficulty.

**Table 4:** The implementation of DTUMOS for various mobility services in various cities.

Spatial region	Mobility system	Description
Seoul metropolitan area	Taxi service	A large-scale mobility system with more than 20,000 vehicles in operation
New York City	Taxi service	Flexible spatial coverage by changing Open Street Map and passenger requests data
City of Chicago	Taxi service	–
Jeju island (South Korea)	Depot-base delivery	Logistics system based on warehouses or depots
Yeouido (South Korea)	Many-to-many pickup & delivery	Applicable to Demand Responsive Transit, food delivery, carpooling

### Case study of decision-making support

This section presents a case study demonstrating the capability of DTUMOS to diagnose the performance of mobility systems and inform policy decisions. The case study focuses on the issue of taxi shortages and increased difficulty in obtaining rides in South Korea following the COVID-19 pandemic. By 2022, the supply of taxis had decreased by over 30% compared to 2019, due to the severe impact of COVID-19 on the taxi industry. However, as COVID-19 recovery progressed and social distancing measures were relaxed after April 2022, there was a surge in taxi demand, particularly during evening and late-night hours, when alternative means of transportation are limited. Therefore, we analyzed the current situation during these hours in the Seoul Metropolitan Area and quantitatively determined the additional taxi supply needed to address the shortage.

In DTUMOS, we modeled the Seoul taxi system for the period from 10:00 PM to 2:00 AM on April 8, 2022. Based on historical pick-up and drop-off data for Seoul taxis, the number of vehicles was set to 24,910 and the number of call requests to 237,626, respectively, as described in Table 5. The scenarios were established as follows: Scenario 1 was based on the data for getting on and off taxis and calling taxis in Seoul between 10:00 PM and 2:00 AM on April 8, 2022, with a supply of 24,910 taxis and passenger demand of 237,626. Scenarios 2, 3, and 4 illustrate the case of increasing the taxi supply by 20%, 50%, and 80%, respectively, compared to Scenario 1. To evaluate the performance of the system, we used indicators such as user waiting time and call failure rate. A call failure was defined as a scenario in which a vehicle was not dispatched or a passenger had to wait for more than 30 min for a vehicle to arrive.

**Table 5:** Changes in Level of Service of taxis by increasing the number of vehicles in Seoul Metropolitan Area during late-night hours (10:00 p.m.–2:00 a.m.): Service level was evaluated by request failures and waiting time of passengers.

Scenario	Supply of taxis	Total call requests	Number of request failures	Request failure rate (%)	Mean waiting time (min)
Scenario 1	24,910	237,626	182,445	76.78	31
Scenario 2	29,892		119,324	51.40	27
Scenario 3	37,365		2598	1.13	9
Scenario 4	44,838		0	0	7

**Figure 4:**
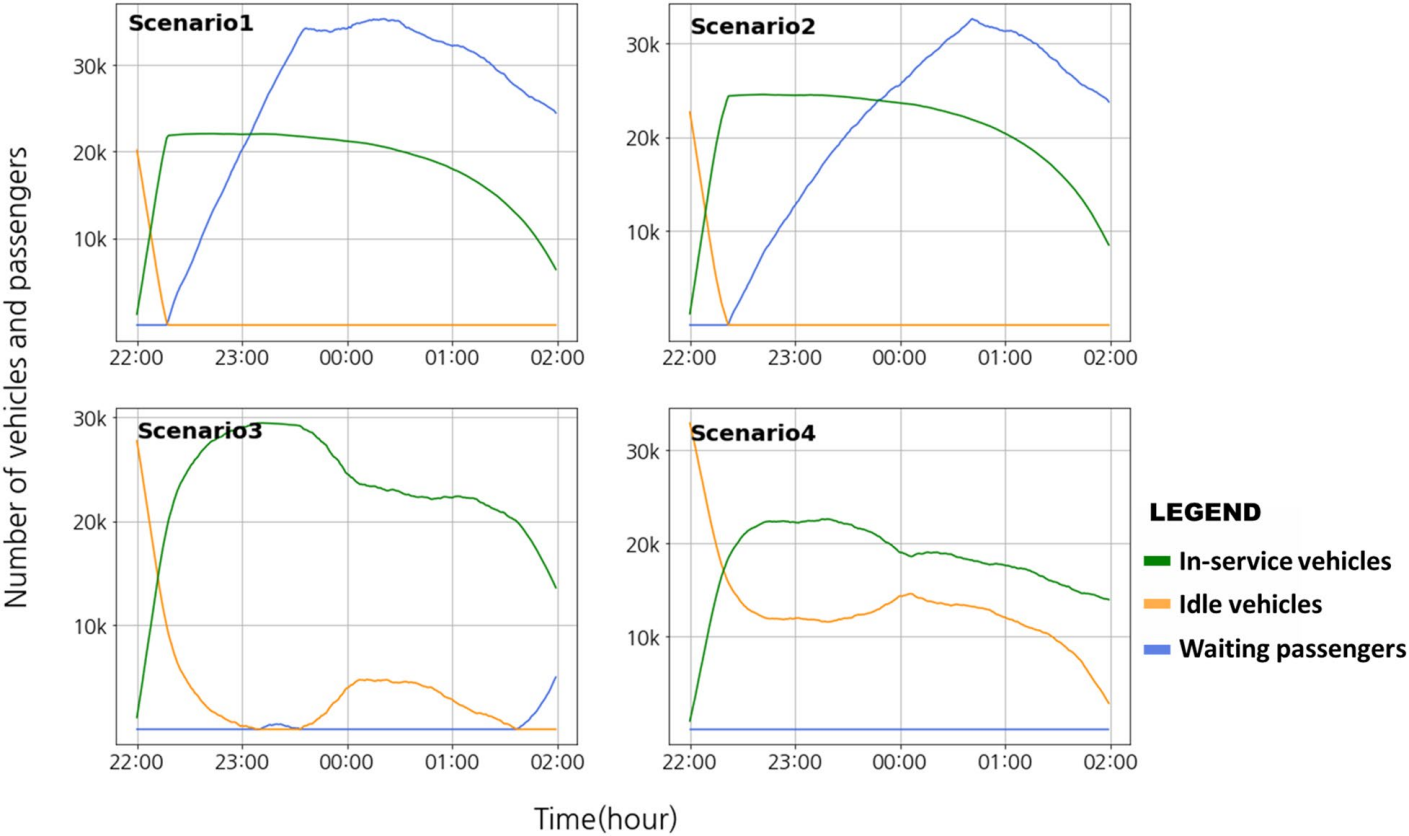
Mobility system operation results by four scenarios in DTUMOS.

Figure 4 illustrates the dynamic changes in the number of vehicles and passengers in operation for each scenario over time. In the case of Scenario 1 and 2, it is evident that the supply of vehicles cannot keep pace with the demand for taxi requests. This results in an increase in the number of passengers waiting for a taxi without being assigned to a vehicle over time. In Scenario 2, the rate of increase of the call queue is reduced compared to Scenario 1, but the supply still falls short, leading to more than 50% of call failures. Conversely, in Scenario 3 and 4, it is noticeable that the number of passengers waiting for a taxi does not escalate, and the system is operating stably. However, in Scenario 4, taxis are oversupplied, leading to a substantial number of idle vehicles (as indicated by the orange line). The results of the simulation conducted through DTUMOS indicate the optimal number of taxis that must be supplied to resolve the taxi shortage. The analysis reveals that a 20% increase in the current supply is not enough to solve the problem, and the supply must increase by approximately 50%.

## Discussion

In the present study, the digital twin for urban mobility operating system, DTUMOS, was introduced. It is implemented as an open-source software to facilitate its application in cities worldwide. The DTUMOOS is lightened by mainly focusing on the essential parts for the implementation of the mobility system. The key to a reliable digital twin for operating mobility systems is to extract the detailed routes of operated vehicles and predict the accurate time it takes for vehicles to drive. We propose a novel architecture that combines an AI-based estimated time of arrival model and vehicle router algorithm to achieve high speed while maintaining accuracy.

The proposed DTUMOS offers distinct advantages in terms of scalability, speed, and visualization when compared to current state-of-the-art mobility digital twins. To the best of our knowledge, this is the first attempt to construct a digital twin capable of covering large-scale metropolitan-level mobility systems. We quantitatively evaluated the performance of DTUMOS using real-world data, and the results showed that it can efficiently handle a mobility system with more than 30,000 vehicles and 200,000 passengers, and provide flexible visualizations. This level of performance is not achievable with existing digital twin platforms for mobility systems.

DTUMOS can be utilized to develop a variety of operation algorithms for mobility systems, including re-balancing empty vehicles, advanced dispatch, and ride-sharing algorithms, dynamic pricing, and fleet size controls.Its lightweight and open-source nature makes it particularly useful for iterative learning methods such as reinforcement learning. Furthermore, DTUMOS can also be exploited to provide quantitative evaluations and guidelines for policies and plans. Decision makers can experiment with different policies and algorithms within the digital twin platform. Through this, they can design policies that are equitable and transparent to all stakeholders related to the mobility services (e.g., platform providers, drivers, and users).

As new and innovative mobility systems are introduced, the concept of Mobility-as-a-Service (MaaS) is emerging and attracting greater attention. Digital twin is also evolved from the conceptual prototype to Digital-twin-as-a-service (DTaaS)^40^. This study serves as a stepping stone to link MaaS and DTaaS. The proposed DTUMOS, which can operate large-scale mobility services in a virtual environment, can provide a test bed for operating mobility services and can directly help various organizations and companies provide reliable MaaS with a high level of service in the real world.

Some limitations are worth noting. Most mobility services do not operate in isolation within a city, and instead interact with various transportation systems. Therefore, it is necessary to incorporate existing transportation systems, such as buses and subways, into the digital twin to enable citizens to transfer between different modes of transportation. To this end, it is required to embed data such as existing bus routes and subway networks in the digital twin and to develop algorithms for modeling people’s travel patterns and mode choices. These are areas of future research.

### Data and code availability

For reproducible and transparent research, we opened all codes and outputs of the research at here. The raw data used in the case study could not be disclosed due to privacy issues, but we released the simulation data for one day where random noise was added.
